# Incident-angle dependent *operando* XAS cell design: investigation of the electrochemical cells under operating conditions at various incidence angles

**DOI:** 10.1039/d0ra09579f

**Published:** 2021-02-04

**Authors:** Seval Gunduz, Dhruba J. Deka, Jaesung Kim, Michael Wilson, Mark Warren, Umit S. Ozkan

**Affiliations:** William G. Lowrie Department of Chemical and Biomolecular Engineering, The Ohio State University Columbus Ohio 43210 USA ozkan.1@osu.edu gunduz.3@osu.edu; Dept. of Physics, Illinois Institute of Technology Chicago Illinois 60616 USA

## Abstract

An *operando* characterization of electrode materials under electrochemical reaction conditions is important for their further development. X-ray absorption spectroscopy (XAS) presents a unique opportunity in this regard as the absence of a vacuum chamber in this technique makes it possible to collect spectroscopy data using user-designed *operando* cells. In the current study, the design and performance of an *operando* XAS cell are evaluated for characterizing solid oxide electrolysis cell working electrodes under a reaction environment that mimics high-temperature ammonia production conditions from H_2_O and N_2_. Sr_2_FeMoO_6−*x*_N_*x*_ (SFMON)-type double perovskite oxides were used as the cathode materials in these experiments. The *operando* cell contained a sample stage with a turnable head so that XAS data can be collected at different angles between the electrode and the X-ray beam with an accuracy of 0.5°. The mechanism to adjust the angle of incidence of the beam on the sample allows control over the depth of penetration of the X-ray photons into the electrode. At low angles, it becomes possible to collect surface sensitive data, which is of great importance as the electrochemical processes are believed to take place on the surface of the electrodes. Sr K-edge and Fe K-edge XAS collected at 2° and 45° angles showed that these the oxidation state changes occurring in these elements are different in the near-surface region compared to the bulk of the electrode. Such an ability to distinguish between the surface and bulk properties of the electrode during real reaction environment will help to understand the underlying phenomena better, which will enable electrode design targeted towards the reactions of interest.

## Abstract 2

Bias and time-dependent changes in the oxidation state and the atomic environment of the atoms of a working electrode occur on the gas/electrode interface.

## Graphical abstract

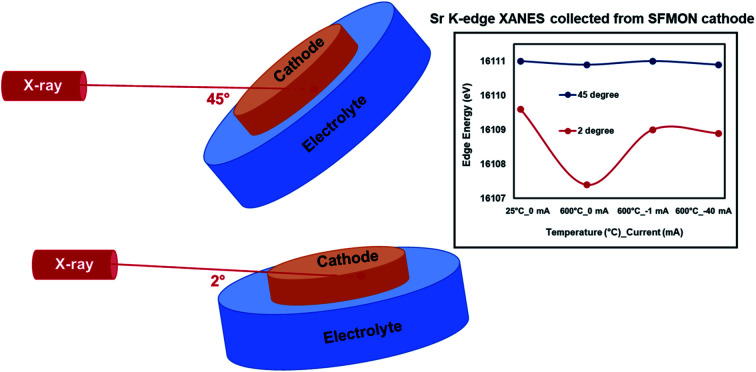

## 1. Introduction

The progress in the development of electrochemical cells depends on a fundamental understanding of the electrochemical reaction mechanisms, ionic and electronic conduction routes and the behavior of the atoms present in the electrode structure under real-electrochemical conditions. Since the high- and intermediate-temperature solid-state electrochemical reaction conditions involve elevated temperatures and electrical potential application under reducing or oxidizing environments, *in situ* and *operando* characterization of the electrochemical cells are not very easy to achieve. X-ray absorption spectroscopy (XAS) technique is one of the best characterization techniques for *in situ* and *operando* studies, since unlike many of the spectroscopy techniques, it does not require vacuum conditions and can be operated under different gas environments at elevated temperatures. XAS is a unique technique for studying the oxidation state and the local structure around the selected elements within a material.^1^ Although in most of the *in situ* XAS studies focused on the electrocatalyst characterization, the model systems, *i.e.* powder form of the electrode materials were used instead of the real electrochemical cells, the recent years have witnessed important progress in the *in situ*/*operando* characterization of electrocatalytic cells as summarized in a recent review paper.^2^

**Fig. 1:**
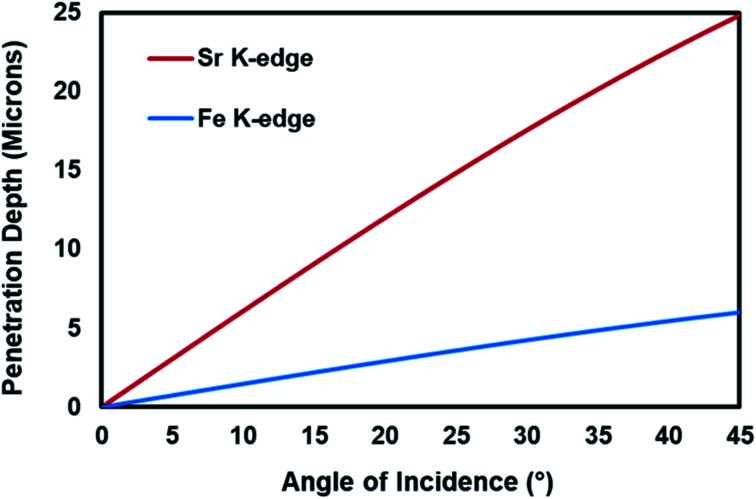
The penetration depth of X-rays for Fe K-edge and Sr K-edge as a function of incident angle.

Another complexity in the investigation of the dynamic changes in the structure of the elements within the working electrode and relating these changes to the performance of the electrochemical cell is that there are different interfaces in an electrode which play different roles in the electrocatalytic activity of the cell and behave differently under real-reaction conditions.^3^ These interfaces are the gas–solid interface, which is the outer most surface of the electrode, and the electrode/electrolyte interface. In order to study the different interfaces and the bulk structure of the working electrode, the penetration depth of the X-rays should be controlled. The penetration depth of the X-rays in XAS is a function of the energy of the X-ray and the angle between the incoming X-ray and the sample.^1,4,5^ The Center for X-ray Optics^6^ provides a tool to estimate the penetration depth of X-rays as a function of energy and the angle of incidence. As shown in Fig. 1, for Fe K-edge (7112 eV) and Sr K-edge (16 105 eV), the penetration depth increases with increasing incident angle and the penetration depth is higher for the element whose edge energy is higher.

The incident-angle dependent X-ray methods have grown rapidly in recent years. The combination of incident-angle dependent methods and X-ray absorption technique allows to obtain detailed structural information of materials as a function of depth. Following the first study conducted by Parratt in 1954,^7^ that suggested the potential of grazing incidence method (low incidence angle) for the structural studies of surfaces, the incident-angle dependent XAS has been used to investigate variety of surface and interface systems.^3,8–12^ Relevant to the present study, Orikasa *et al.*^13^ studied the structural changes of a La_0.8_Sr_0.2_CoO_3−δ_-type solid oxide fuel cell (SOFC) cathode from the surface to the bulk using the angle-resolved (incident angle dependent) XAS technique under real electrochemical reaction environment and electrical bias. Although their experimental design allows the investigation of SOFC cathodes under real-reaction environment, it cannot be used for other type of electrochemical systems, since the gas environment of the working electrode cannot be controlled using that system.

In the present study, a new *operando* incident-angle dependent (IAD)-XAS cell design is presented and discussed. The cell is capable of studying the electrochemical cells under real-reaction environment, as well as the investigation of different interfaces (depths) on the working electrode. The *operando* IAD-XAS cell design combines the benefit of the *in situ* XAS cells' ability to mimic the real-reaction environment and the angle-resolved XAS cells' unique ability of controlling the analysis depth of the sample.

## 2. Design and experimental details

In this part of the paper, the details about the *operando* IAD-XAS cell design, the preparation of the button cells used in the XAS experiments and *operando* XAS data collection at two different incidence angles, *i.e.* 45° and 2°, are presented.

### 2.1. Incident-angle dependent *operando* XAS cell design

The *operando* IAD-XAS cell was designed by our research group in order to be able to characterize the electrochemical cells used in various electrocatalysis studies such as high-temperature electrolysis of CO_2_ and H_2_O and high-temperature electrocatalytic synthesis of NH_3_ from N_2_ and H_2_O, which are the current projects that our electrocatalysis research team is focused on.

The *operando* IAD-XAS cell was fabricated in the machine shop that belongs to the Department of Chemical and Biomolecular Engineering at the Ohio State University by the laboratory supervisor.

**Fig. 2:**
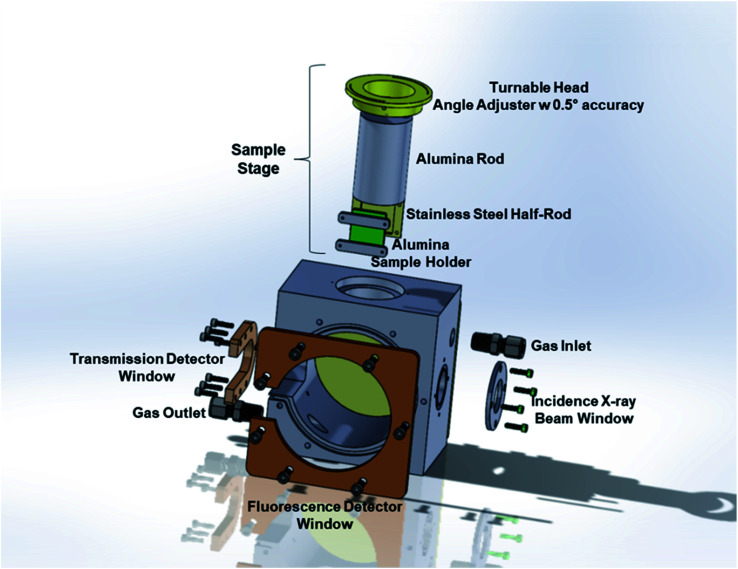
Schematic representation of the *operando* incident-angle dependent XAS cell.

The *operando* IAD-XAS cell has two main parts: the main body and the sample stage. A schematic representation of the cell is shown in Fig. 2.

The main body of the XAS cell is made of stainless steel. There are two X-ray windows on the opposite sides of the cell for the incident and transmitted beams. The large window placed at the front side is used for the detection of the fluorescence beam. One of the advantages of the present design is that it allows the detection of the scattered beam from the sample using both transmission and fluorescence detectors. The stainless-steel part between the transmission and fluorescence windows was taken out in order to be able to collect the most intense scattered beam using the fluorescence detector placed at the optimum position/angle for various incident angles. The removable front and back plates ensure practical access to the sample holder to replace the button cells in between each experiment. The incident beam and the transmission and the fluorescence detection windows are sealed with Kapton® tape (with a thickness of 3 mils = ∼0.08 mm in order to minimize the absorption by the tape) and high-temperature resistant O-rings. The leak-check tests performed at a temperature range of 25–700 °C showed that the cell is leak-free within this temperature range at atmospheric pressure.

**Fig. 3:**
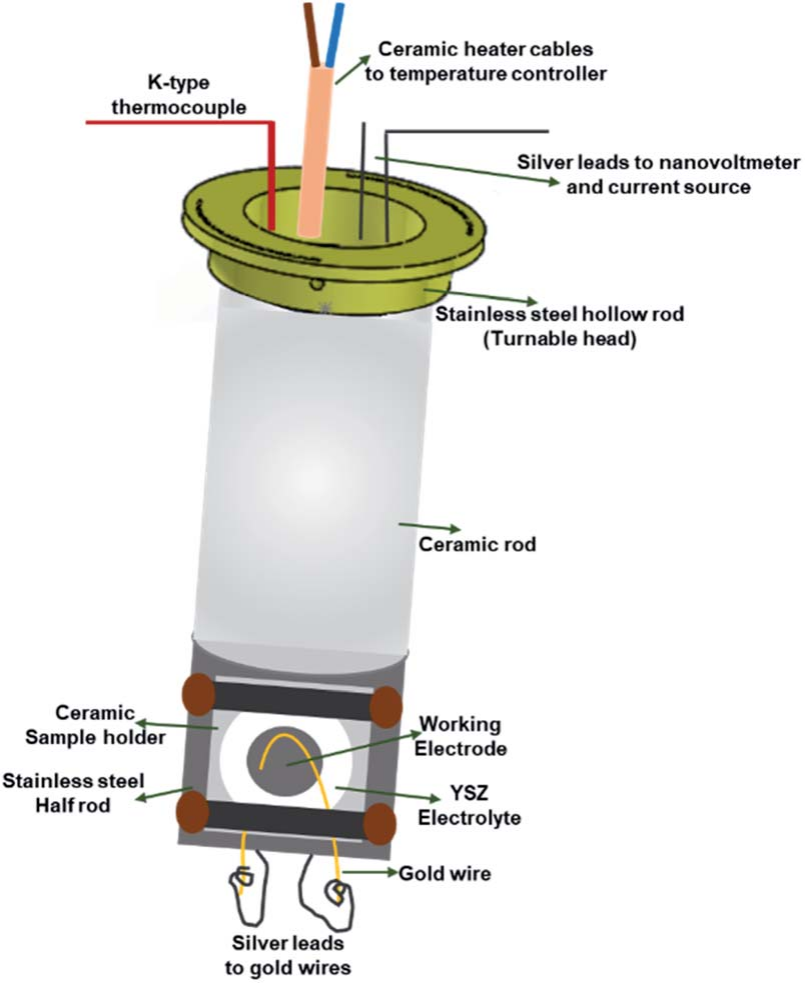
Schematic representation of the *operando* incident-angle dependent XAS cell sample stage.

The sample stage (Fig. 3) has two main parts; (1) alumina rod that houses a ceramic micro-heater, a K-type thermocouple and silver leads, and (2) stainless steel part which supports the alumina sample holder and ensures a good heat transfer between the ceramic heater and button cells.

The ceramic rod is mounted inside and screwed to a stainless-steel hollow rod. The hollow rod that supports the sample stage is turnable, therefore the angle between the incident X-ray beam and the sample can be adjusted. The angle scale present on the top side of the main body helps to adjust the angle and the sample stage is secured at a particular angle using pins. The silver leads (current collectors) are guided through the hollow ceramic micro-heater and move with the stage whenever the angle is changed. This design ensures that all the parts inside the stage move together which eliminates extra force on the electrical leads or a damage to the heater or the thermocouple. The bottom part of the sample stage, from where the silver leads are taken out to attach to the gold wires on the electrodes, is sealed with a ceramic plate using a high-temperature ceramic seal. The silver leads taken out from the top part of the cell are connected to the Keithley current source and nanovoltmeter to apply controlled current and measure the corresponding voltage, respectively.

The ceramic sample holder is placed inside the carved stainless-steel part of the sample stage. In this part, both the stainless steel and the ceramic materials are used to maintain a uniform heat distribution and a metal-free surface to prevent short-circuit, respectively.

### 2.2. Button cell preparation for XAS studies

The button cells used in this study to test the applicability of the present cell design for angle dependent (depth profiling) analysis are composed of a YSZ electrolyte and LSM-YSZ on both sides of the solid electrolyte to serve as electrode and interlayer for counter electrode side and working electrode side, respectively. YSZ and LSM-YSZ are the abbreviations for 8 mol% yttria-stabilized zirconia and 1 : 1 wt% mixture of La_0.8_Sr_0.2_MnO_3_ and 8 mol% yttria-stabilized zirconia, respectively.

In the present study, two different working electrodes were tested in order to evaluate the efficiency of the *operando* IAD-XAS cell. In order to investigate if the new cell design is efficient for both XANES and EXAFS studies, La_0.7_Sr_0.2_Ni_0.2_Fe_0.8_O_3_ (LSNF)-type perovskite oxide working electrode was used. For the *operando* XANES studies, Sr_2_FeMoO_6−*x*_N_*x*_ (SFMON)-type double perovskite oxynitride working electrode was tested. The synthesis methods of LSNF and SFMON can be found in ref. 14 and 15, respectively.

For a button cell fabrication, first a circular disc was cut from a tape-casted YSZ sheet and densified at 1450 °C in air. After the densification, the thickness of the YSZ disc was 125 μm with a 2.54 cm diameter. The commercial LSM-YSZ powder (Nexceris Materials) mixed with an ink vehicle (Nexceris Materials) in a 60 : 40 wt ratio was then screen-printed on to the both sides of the densified electrolyte and fired at 1200 °C to facilitate a good contact between the electrolyte and the electrodes. The post-firing thickness of the electrodes was 25 μm. Following the firing of LSM-YSZ electrode layers, either LSNF or SFMON slurry was brush-painted on one of the LSM-YSZ layers to serve as the working electrode with 15–25 μm thickness and dried in an oven at 120 °C overnight. Once the button cell was ready, gold wires (0.1 mm O.D., 99.99% trace metal basis, Sigma Aldrich) were attached on both electrodes using high temperature conductive gold paste (ESL Electroscience) and dried at 100 °C for 1 h in air. The gold leads act as current collectors together with the silver wires in the *operando* IAD-XAS cell.

### 2.3. XAS data collection

In the present study, two sets of experiments were conducted in order to evaluate the *operando* IAD-XAS cell.

*In the first set of experiments*, two different *operando* XAS cell designs were used. The first design was a custom-made quartz tube with a conical tube connected to it at the center (F-reactor). The details about the F-reactor design can be found in our earlier publication.^14^ The button cell with LSNF working electrode was placed at the center of the F-reactor at 45° angle to the incident X-ray beam. The ends of the quartz tube were connected to custom-made vacuum fittings which contain gas inlet/outlet and sealed routes for the electrical leads. The fluorescence (Lytle) detector was placed in front of the conical tube at 90° with respect to the incidence beam in order to minimize the elastic scattering contribution (for a synchrotron X-ray radiation emitted from a bending magnet, *i.e.* BM beamlines).^16^ The second cell was the incident-angle dependent *operando* XAS cell described earlier. The same LSNF button cell was clamped onto the sample holder of the sample stage (Fig. 3) which was placed inside the main body (Fig. 2). The angle between the working electrode and the incoming X-ray beam was set to 45° by rotating the movable head on which the angles are marked with 0.5° intervals. The Lytle detector was placed in front of the fluorescence detector window at 90° with respect to the incidence beam in order to minimize the elastic scattering contribution. The main aim of this set of experiments was to evaluate and compare the noise levels of the EXAFS regions obtained with these two *operando* XAS cell designs. In accordance with this aim, the Fe K-edge region of the LSNF electrode inside both the cell designs was studied.

*In the second set of experiments*, the applicability of the *operando* IAD-XAS cell to the *operando* characterization of the working electrodes under electrochemical reaction environments at various grazing incidence angles was studied. The main aim of this set of experiments was to investigate the changes in the oxidation state of the atoms in the electrode materials from different interphases by changing the angle between the incident X-ray beam and the sample. In the present study, the SFMON working electrode was studied in an environment that mimics the high-temperature electrocatalytic ammonia synthesis from N_2_ and H_2_O which is one the projects that the authors are currently working on. The button cell with SFMON working electrode was clamped onto the ceramic sample holder placed inside the main body. The gold wires placed on the electrodes were attached to the silver leads which were attached to the Keithley current source and nanovoltmeter to apply current and measure the corresponding voltage, respectively. A 50 sccm of gas stream that contains 3% H_2_O and N_2_ in balance was sent to the *operando* IAD-XAS cell in order to create a similar environment with the high-temperature electrocatalytic ammonia synthesis. The temperature of the cell was set to 600 °C. In this set, two different incidence angles were tested, *i.e.* 45° (standard XAS geometry in fluorescence mode) and 2° (grazing incidence angle), in order to collect XANES data from bulk of the electrode and from a layer close to the gas-electrode interlayer (surface-sensitive) where the electrocatalytic reaction is believed to occur, respectively. The Sr K-edge and the Fe K-edge XANES data was collected in order to investigate the changes in the oxidation states of A-site and B-site atoms during electrochemical process. Prior to the XAS experiments at both edge energies, the size of the beam was adjusted. The size of the beam is an important factor that can affect the quality of the data collected. Since the button cell area is limited, the size of the beam was minimized for both Sr and Fe K-edge energy beams in such a way that the scattering from the surroundings of the working electrode was eliminated and a respectful edge-jump was observed for both elements.

## 3. Results and discussion

### 3.1. XAS data collection

As described in Section 2.3, two different *operando* XAS cells were used to collect Fe K-edge XAFS data of LSNF working electrode at room temperature. A Lytle detector was used to collect XANES and EXAFS data in fluorescence mode. The angle between the incoming X-ray beam and the button cell was kept at 45° which is the standard XAS geometry in fluorescence mode for both F-reactor and *operando* cell. X-ray absorption spectrum of Fe metal foil was taken before and after each measurement in order to calibrate the data for any shifts in the X-ray energy. The data obtained from the Fe foil and the button cells placed in the F-reactor and in the *operando* cell were processed using the Athena software.^17^ The same parameters were used in order to be able to compare the performances of the *operando* cell designs, *i.e.* F-reactor and IAD-XAS cell.

**Fig. 4:**
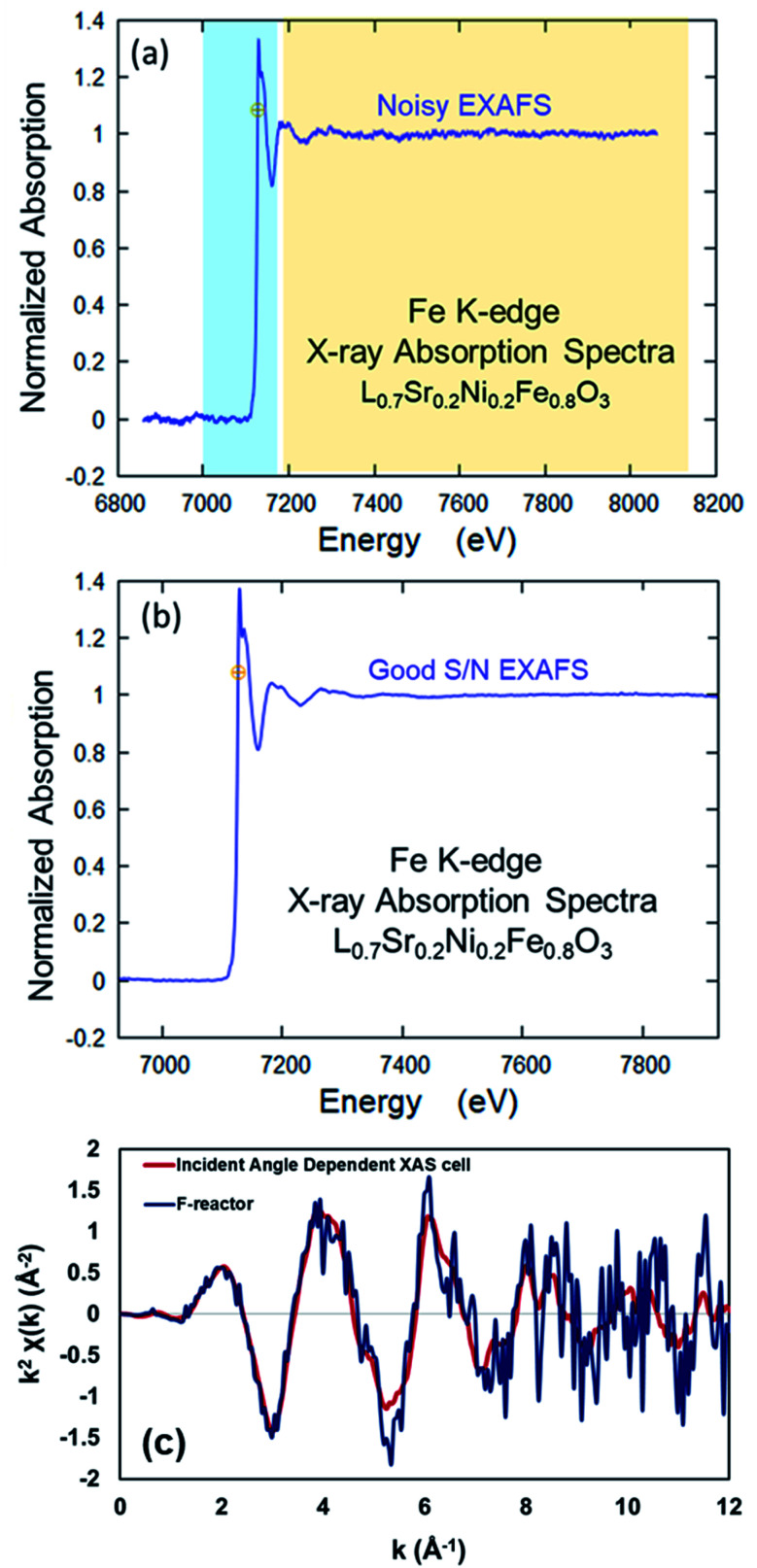
Comparison of the XAS data collected from LSNF at room temperature using (a) F-reactor, (b) the *operando* IAD-XAS cell and (c) comparison of the data collected in *k*-space.

As seen in Fig. 4, the XAS data collected from the electrochemical cell with LSNF working electrode using the incident-angle dependent *operando* cell has a better signal-to-noise ratio (especially in the EXAFS region) compared to the data collected using the F-reactor.

The data collected using both XAS cells was also plotted in *k*-space (Fig. 4c) in order to compare the quality of the data. As seen, the data collected using the F-reactor is very noisy; especially in the high *k* region (6–12 Å) of the spectrum, the signal is swamped by the noise which makes the Fourier transforms difficult to interpret.

The difference in the quality of the data collected using these two XAS cells is conceivably due to the difference in the distances between the sample and the fluorescence (Lytle) detector. Due to the presence of the conical tube in the F-reactor design, the fluorescence detector cannot be placed close to the sample which leads to the lower count of fluorescent photons to be detected, therefore causes noisy data in the EXAFS region.

These results suggest that the *operando* IAD-XAS cell would be a better choice for the *in situ* experiments under current conditioning and real electrochemical reaction environment.

### 3.2. *Operando* XANES analysis of SFMON electrode under high-temperature electrocatalytic ammonia synthesis environment at two incident beam angles: 45° and 2°

The main aim of this part of the study is to evaluate the performance of the incident-angle dependent *operando* XAS cell for the investigation of the bulk and near-surface properties of the working electrode under high-temperature electrocatalytic NH_3_ synthesis environment during current-conditioning. As mentioned in the Introduction section, there is a direct correlation between the penetration depth of the X-rays and the energy of the beam and the incident angle. Since for each atom, the absorption/excitation energy is fixed, we can regulate the penetration depth of the X-ray by changing the incidence angle between the incoming X-ray and the sample. In this study, two different incidence angles were used, *i.e.* 45° and 2°, to investigate the changes in the oxidation state of Sr (A-site atom) and Fe (B-site atom) of the SFMON electrode with heating and current-conditioning in the bulk and at the near-surface, respectively. In addition to the 45° incidence angle orientation, which is the standard geometry for the XAS measurements in the fluorescence mode, 2° incidence angle orientation was also studied since it is believed that the electrochemical reactions mostly occur on the gas/electrode interface (surface of the electrode).^3^ It is a known fact that as the incidence angle decreases the penetration depth of the X-rays also decreases.^1,4,5^ If the incidence angle is fixed to a small value such as 2°, the XAS measurement can become more surface sensitive.

Based on the preliminary electrocatalytic activity results of the SFMON working electrode for high-temperature electrocatalytic NH_3_ production at 600 °C, 3 different current values were chosen: 0 mA (Open Circuit Voltage-OCV condition), 1 mA and 40 mA. While no NH_3_ formation was observed at OCV, NH_3_ was detected at 1 mA and 40 mA, with the NH_3_ formation rate being higher at 1 mA than 40 mA.

**Fig. 5:**
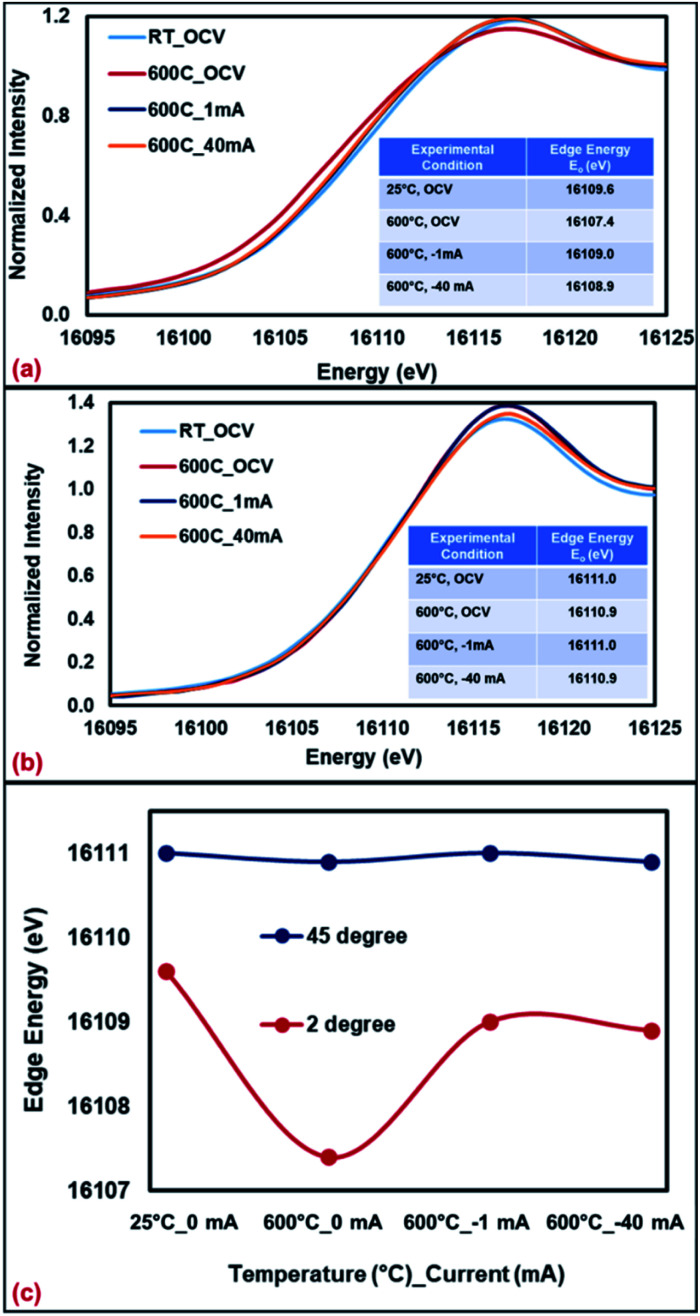
Sr K-edge XANES collected from SFMON electrode at room temperature (OCV) and 600 °C (OCV, 1 mA and 40 mA) at (a) 2°, (b) 45° and (c) comparison of the edge energies.

The Sr K-edge XANES of SFMON electrode under 3% H_2_O/N_2_ environment under OCV condition and current application (1 mA and 40 mA) collected at room temperature and 600 °C are shown in Fig. 5a and b, with the angle of incidence being 2° and 45°, respectively.

It can be seen from the Fig. 5 and the inset tables that when the incident angle is 2° (surface-sensitive geometry), the oxidation state of the A-site atom Sr significantly decreases as the temperature is raised from RT to 600 °C under OCV conditions, which suggests the reduction of the Sr ions on the near-surface. However, when the potential is applied (current-conditioning), the oxidation state of the Sr ions on the near-surface increases which indicates the re-oxidation of Sr ions under current-conditioning. When the data was collected at 45° (standard geometry), no changes on the oxidation state of the Sr ions in the bulk with heating or current-conditioning were observed. The edge energy of Sr atoms remained the same throughout the experiment. These results support the assertion that the bias and time-dependent changes in the oxidation state and the atomic environment of the atoms of a working electrode occur on the gas/electrode interface (at the upper surface of the electrode) rather than in the bulk of the working electrode.

**Fig. 6:**
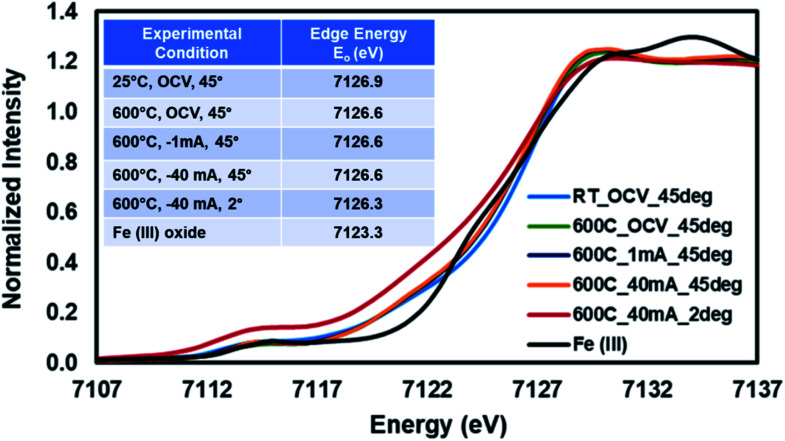
Fe K-edge XANES collected from SFMON electrode at room temperature (OCV) and 600 °C (OCV, 1 mA and 40 mA) at 45° and 600° (40 mA) at 2°.

The same experiment was repeated using a fresh SFMON button cell to study the behavior of the B-site Fe atom under the same environment and current-conditioning. Fig. 6 shows the Fe K-edge XANES data collected at room temperature (OCV), 600 °C (OCV, 1 mA and 40 mA) at the incident angle of 45° and also at 600 °C (40 mA) at the incident angle of 2° in order to check if there is a difference between the oxidation states of Fe in the bulk and at the near-surface of the electrode under the same conditions.

One interesting observation is that the oxidation state of the Fe in the SFMON sample is higher than +3 which suggests that the SFMON-type double perovskite oxynitride sample contains Fe^4+^/Fe^3+^ redox couples which lead to an oxidation state of more than +3 for iron. As seen from the Fig. 6 inset table, there are no significant changes in the oxidation state of iron atoms with heating and current-conditioning. The oxidation state of the iron collected at 2° incident-angle, 600 °C and −40 mA (*E*_o_ = 7126.3 eV) is slightly lower than that of iron collected at room temperature (*E*_o_ = 7126.9 eV) and at 45° incident-angle, 25 °C/600 °C, and OCV/−1 mA/−40 mA (*E*_o_ = 7126.6 eV). Unlike the case for the Sr ions, the current-conditioning causes a more reduced state for the Fe ions instead of causing a re-oxidation. This phenomenon can be explained by the iron atoms' high affinity for interaction with hydrogen. As a result of the current-conditioning, water electro-reduction happens which produces H_2_. The amount of the hydrogen produced during the water electro-reduction increases with the increasing current. Since the production rate of NH_3_ cannot compete with the production rate of H_2_, the unreacted H_2_ starts to accumulate on the surface of the electrode which affects the oxidation state and the atomic environment of the Fe atoms more than that of Sr ions on the gas/electrode interface. The difference between the oxidation states of Fe in the bulk and on the near-surface of the electrode is not as prominent as in the case of Sr due to the fact that, as mentioned in the previous sections, the penetration depth of the X-rays also depends on the energy of the incoming X-ray beam. Since the Fe K-edge (7112 eV) energy is lower than the edge energy of Sr (16 104.6 eV), the penetration depth of the incoming X-rays is significantly lower for the experiments conducted at the Fe K-edge. Therefore, the oxidation state of the iron did not show significant difference at 2° and 45° incident angles.

These preliminary results show that the *operando* IAD-XAS cell is suitable for the *operando* characterization of the working electrodes under electrochemical reaction conditions and current-conditioning. The cell design is sensitive enough to study the bias- and time-dependent changes in the oxidation state and the atomic environments of the atoms in the electrode material both in the bulk and on the near-surface (gas/electrode interface).

## 4. Conclusions

In the present study, a new incident-angle dependent *operando* XAS cell designed for the investigation of the bulk and near-surface properties of the working electrodes under electrochemical reaction environment and current-conditioning.

The comparison of the XAS data collected using the F-reactor and the new *operando* IAD-XAS cell shows that the latter design provides data with better signal-to-noise ratio, especially in the EXAFS region compared to the data collected using the former.

The most important advantage of the *operando* IAD-XAS cell design is that it allows us to investigate the atomic environment and the oxidation state of the atoms in the electrode material at different sample depths by changing the angle between the incident X-ray beam and the electrode.

The preliminary results suggest a potential use of this type of an IAD-XAS cell for the investigation of working electrodes for different electrocatalytic reactions such as CO_2_ and H_2_O electrocatalytic reduction, NO_*x*_ reduction, SOFC cathode applications and electrocatalytic NH_3_ production from N_2_ and H_2_O as described in the present study.

## Conflicts of interest

There are no conflicts to declare.

## Supplementary Material
